# T-cell exhaustion in chronic hepatitis B infection: current knowledge and clinical significance

**DOI:** 10.1038/cddis.2015.42

**Published:** 2015-03-19

**Authors:** B Ye, X Liu, X Li, H Kong, L Tian, Y Chen

**Affiliations:** 1 State Key Laboratory for Diagnosis and Treatment of Infectious Diseases, Collaborative Innovation Center for Diagnosis and Treatment of Infectious Diseases, The First Affiliated Hospital, College of Medicine, Zhejiang University, Hangzhou, People's Republic of China

## Abstract

Hepatitis B virus (HBV) infection is the major cause of inflammatory liver disease, of which the clinical recovery and effective anti-viral therapy is associated with the sustained viral control of effector T cells. In humans, chronic HBV infection often shows weak or absent virus-specific T-cell reactivity, which is described as the ‘exhaustion' state characterized by poor effector cytotoxic activity, impaired cytokine production and sustained expression of multiple inhibitory receptors, such as programmed cell death-1 (PD-1), lymphocyte activation gene-3, cytotoxic T lymphocyte-associated antigen-4 and CD244. As both CD4^+^ and CD8^+^ T cells participate in the immune responses against chronic hepatitis virus through distinct manners, compelling evidences have been proposed, which restore the anti-viral function of these exhausted T cells by blocking those inhibitory receptors with its ligand and will pave the way for the development of more effective immunotherapeutic and prophylactic strategies for the treatment of chronic infectious diseases. A large number of studies have stated the essentiality of T-cell exhaustion in virus-infected diseases, such as LCMV, hepatitis C virus (HCV), human immunodeficiency virus infections and cancers. Besides, the functional restoration of HCV- and HIV-specific CD8^+^ T cells by PD-1 blockade has already been repeatedly verified, and also for the immunological control of tumors in humans, blocking the PD-1 pathway could be a major immunotherapeutic strategy. Although the specific molecular pathways of T-cell exhaustion remain ambiguous, several transcriptional pathways have been implicated in T-cell exhaustion recently; among them Blimp-1, T-bet and NFAT2 were able to regulate exhausted T cells during chronic viral infection, suggesting a distinct lineage fate for this sub-population of T cells. This paper summarizes the current literature relevant to T-cell exhaustion in patients with HBV-related chronic hepatitis, the options for identifying new potential therapeutic targets to treat HBV infection and highlights priorities for further study.

## Facts

- Chronic hepatitis B is a heterogeneous and refractory disease with poor prognosis as well as limitations including expensive cost, viral resistance and toxicity with ongoing anti-viral therapy.
- Patients with chronic HBV infections are usually characterized by a population of exhausted T cells, which have weak virus-specific T-cell responses during chronic HBV infection, impeding the clearance of virus and recovery from hepatitis.
- The mechanism of exhausted T cells in persistent infections such as LCMV and cancers have been well described, and related antibody blockade treatments have been applied, which have achieved evident outcomes. However, there is a significant lack of the underlying mechanisms of CD8^+^ and CD4^+^ T-cell exhaustion.
- Recent progresses in the exploration of exhausted T cells during chronic HBV infection have provided novel insight for the possibility of immunotherapy for this disease.

## Open Questions

- As the effector function of T cells have been impaired during chronic HBV infection, we wonder whether and how the function of exhausted T cells can be restored to regain their anti-viral ability?
- Although previous studies mainly focus on the CD8^+^ exhausted T cells, more and more attention have been paid on CD4^+^ exhausted T cells; thus, we propose our question whether CD4^+^ exhausted T cells have similarly important roles in chronic HBV infection?
- Why does the blockade treatment restore the function of exhausted T cells only in partial patients, and why is the therapeutic outcome distinct among different research groups?
- Can the combination of several antibodies achieve better effect on the restoration of exhausted T cells in the treatment of chronic HBV?
- Whether the exhausted T cells in chronic HBV infection were regulated by specific transcriptional pathways?

Hepatitis B virus (HBV) is the most prevalent virus that leads to liver injury and inflammation. During the acute phase of infection, effective T-cell response for viral clearance of HBV infection is characterized by active and sustained multiepitope-specific CD4^+^ and CD8^+^ T-cell responses. CD4^+^ T cells are provided with the ability to target HBV core antigen epitopes and produce Th-1-type cytokines such as interferon-*γ* (IFN-*γ*) and tumor necrosis factor-*α* (TNF-*α*), as well as the ability to induce and maintain successful CD8^+^ T-cell responses.^1, 2, 3, 4^ The anti-viral functions of HBV-specific CD8^+^ T cells were verified in experimentally infected chimpanzees, indicating that CD8^+^ T cells are the major immune cells contributing to the clearance of HBV.^5, 6^ Contrarily, chronic HBV infection fails to mount such an efficient innate and adaptive immune response to the virus, resulting in a lengthy and complicated combat between immune clearance and tolerance with respective immunological characteristics and clinical manifestation as summarized in Table 1. It is currently not understood how the establishment of chronicity leads to a state of relative collapse of virus-specific T cells.

**Figure 1:**
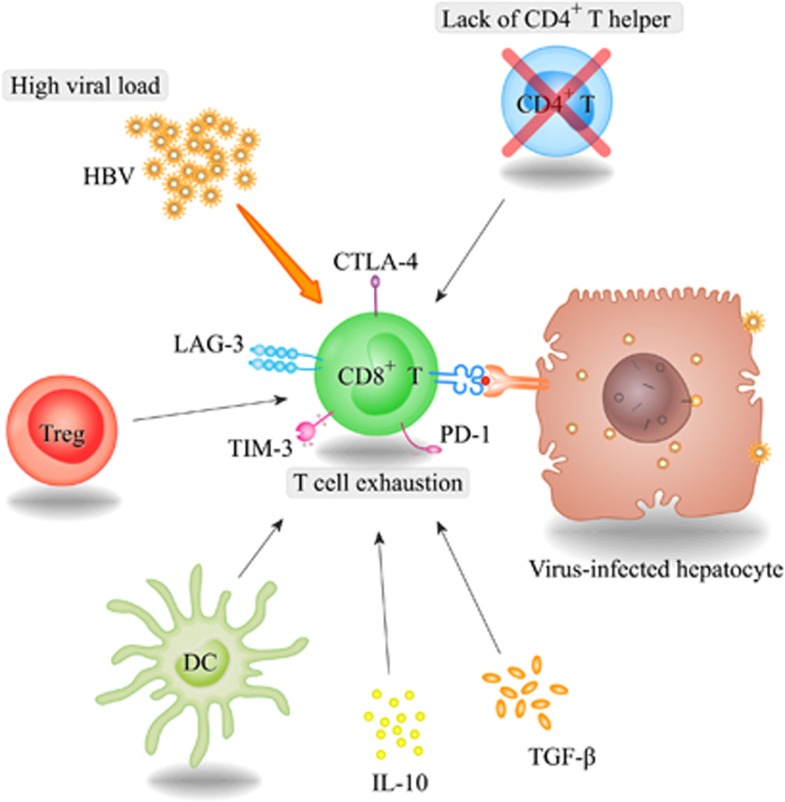
Mechanisms contributing to the exhaustion of HBV-specific CD8^+^ T cells. There are several mechanisms involved in T-cell exhaustion during chronic HBV infection, including high viral (or antigen) load, loss of CD4^+^ T-cell help, suppressive cytokines IL-10 and TGF-*β* and DCs, as well as Treg cells, which are the major sources of the immunosuppressive cytokines IL-10 and TGF-*β*. All of these factors were able to promote the exhaustion of T cells during chronic HBV infections.

In recent years, investigations of the mechanisms underlying these impaired T cells in patients who develop chronic HBV infection have been on the increase. It has been established that several mechanisms may contribute to the dysfunction of HBV-specific T cells, such as continuously high viral load and high antigen levels, suppressive cytokines including interleukin-10 (IL-10) and transforming growth factor-*β* (TGF-*β*), dendritic cells (DCs) and regulatory T (Treg) cells, which were able to result in a progressive loss of T-cell function and cause HBV-specific T cells to become exhausted^7, 8^ (Figure 1). Such ‘exhausted T cells' have been correlated with a hierarchical dysfunction of their proliferative capacity, effector function (impaired cytokine production) and increased apoptosis.^9, 10, 11^ Emerging evidences have indicated that during chronic infection, exhausted T cells develop not only with the change of phenotype but also with the distinction of function; finally, these dysfunctional T cells progress to apoptosis because of the defect of differentiating into memory T cells.^12^ Functional exhaustion of T cells was not only observed in chronic HBV infection but also has been confirmed in cancers owing to the ability of developing high antigen and immunosuppressive environment. In cancer environment, tumor-reactive T cells have also shown an upregulation of inhibitory molecules, such as programmed cell death-1 (PD-1) and cytotoxic T-lymphocyte-associated antigen-4 (CTLA-4), which resemble T-cell exhaustion in chronic viral infection.^13, 14^ Specifically, studies on human metastatic melanoma have shown that both CD8^+^ and CD4^+^ tumor-infiltrating lymphocytes had significantly higher expression of PD-1 and CTLA-4, whereas PD-1 blockade enhanced the frequency of cytokine-producing cells.^15, 16^ Furthermore, another clinical study stated that blocking the PD-1 immune checkpoint in patients with treatment-refractory solid tumors was well tolerated and associated with the evidence of antitumor activity.^17^ For other virus-infected diseases such as LCMV (lymphocytic choriomeningitis), HCV (hepatitis C virus) and HIV (human immunodeficiency virus) in mice and humans, there were numerous evidences supporting that restoring the function of intrahepatic T-cell function can be achieved by blocking single or a combination of inhibitory pathways.^18, 19, 20, 21, 22^ Recent efforts have proposed that specific transcription factors such as T-bet were associated with T-cell exhaustion, which were supported by the evidence that T-bet directly repressed transcription of the gene encoding PD-1, and high T-bet expression sustained exhausted CD8^+^ T cells in mice infected with LCMV,^23^ reminiscent of its role in sustaining exhausted CD4^+^ T cells.^24^ Based on the above researches, more and more attention have been focused on the mechanism and clinical significance of T-cell exhaustion in HBV infection. In this paper, we will draw upon the most up-to-date available data to understand the behavior of T cell exhaustion during chronic HBV infection in humans, and meanwhile, we will characterize several potential immunotherapeutics upon the manipulation of inhibitory molecules for the future treatment of chronic HBV infection.

## CD8^+^ T Cells are Identified with Impaired Ability of Proliferation in Chronic HBV Infection

**Figure 2:**
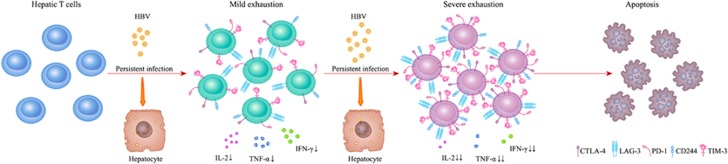
The hierarchical development of T-cell exhaustion during persistent viral infection. In chronic viral infection, T-cell exhaustion is a well-defined state characterized by stepwise and progressive loss of T-cell function. As antigen or viral load increases, the expression of coinhibitory receptors such as PD-1, TIM-3, CTLA-4 and CD244 (2B4) were remarkably increased on the surface of exhausted T cells, which is closely associated with their unresponsiveness. Furthermore, in a hierarchical manner, exhausted T cells lose their proliferative capacity and effector function, including impaired cytokine production such as IL-2, TNF-*α* and IFN-*γ*. Ultimately, in the severe stage of exhaustion, virus-specific T cells can be completely deleted, leading to the loss of virus-specific T-cell responses. The cytokine production is indicated by arrows from decrease (↓) to significant decrease (↓↓)

In chronic infection of human and mice, T-cell exhaustion is a well-defined state characterized by stepwise and progressive loss of T-cell function summarized in Figure 2. As antigen or viral load increases in chronic infection, the expression of coinhibitory receptors such as PD-1, T-cell immunoglobulin domain and mucin domain 3 (TIM-3), CTLA-4 and CD244 (2B4) were remarkably increased on the surface of exhausted T cells, which is closely associated with their unresponsiveness.^25, 26, 27, 28^ The elimination of virus was largely hindered by the reduced number and weak virus-specific T-cell response during persistent infection.^29^ To comprehend the escape and tolerance mechanism of HBV in chronic hepatitis B (CHB) patients, more and more researchers have begun to explore the appearance and essence of CD8^+^ T-cell exhaustion in HBV-infected patients. Recently, the related mechanism analyzed by GeneChip technology revealed that an apoptosis gene *Bim* (Bcl2-interacting mediator) was consistently and significantly expressed in HBV-specific CD8^+^ T cells from CHB patients compared with those in resolved patients; hence, Bim-mediated apoptosis may contribute to the exhausted state of CD8^+^ T cells and impede their response to persist viral replication.^10^

## Exhausted CD8^+^ T Cells Upregulate the Inhibitory Receptors During HBV Infection

As antigen or viral load increases in chronic infection, the expression of coinhibitory receptors, such as PD-1, CTLA-4, CD244 (2B4) and TIM-3, was remarkably increased on the surface of exhausted T cells, which is closely associated with their unresponsiveness.^25, 26, 27, 28^ Correspondingly, blockade of these inhibitory pathways could rescue exhausted virus-specific CD8^+^ T cells by improving T-cell proliferation, cytotoxicity and cytokine production.^20, 26, 30^

## PD-1/PD-L1

Except for defective proliferation, the enhanced expression of some inhibitory receptors may contribute to the CD8^+^ T-cell exhaustion in chronic HBV-infected patients.^31^ Significant finding in CHB patients showed that circulating HBV-specific CD8^+^ T cells were mainly PD-1 positive;^11^ in line with those observations in other different viral infections, the exhausted T cells affected by high viral loads would upregulate their expression of PD-1.^9, 32, 33^

Among all those inhibitory receptors expressed on exhausted T cells, PD-1 was considered as the dominant responsive inhibitory receptor.^34^ As HBV-specific CD8^+^ T cells markedly upregulated the expression of PD-1, more in-depth studies were performed to block the PD-1/programmed death-ligand 1 (PD-L1) interaction in peripheral blood mononuclear cells (PBMCs) isolated from chronic HBV patients, and found that the expansion ability and cytokine secretion of both CD8^+^ and CD4^+^ T cells were partially reversible upon anti-PD-L1 blockage,^11^ suggesting the importance of the PD-1/PD-L1 pathway in exhausted T cells during chronic HBV infections. Simultaneously, several other researches confirmed the significance of the PD-1/PD-L1 pathway on exhausted T cells in chronic HBV-infected patients. The evidences provided by these articles not only showed the reduced effector functions and elevated PD-1 expression on exhausted CD8^+^ T cells but also verified the blockade of the PD-1/PD-L1 pathway could result in increased proliferation and IFN-*γ* and IL-2 production of CD8^+^ T cells specific for HBV.^35^ However, the effect of blocking PD-1/PD-L1 is remarkably different between intrahepatic T cells and peripheral T cells, as the functional recovery of exhausted HBV-specific T cells were more pronounced for intrahepatic T cells than T cells from the periphery after neutralization of the PD-1/PD-L1 pathway.^35^ Taken together, it is well defined that high antigen concentrations and persistent stimulation of HBV can promote HBV-specific T-cell exhaustion by affecting the phenotype and function of peripheral and intrahepatic T cells through the PD-1/PD-L1 pathway, but the exact mechanism remains to be elucidated as the partial recovery of some HBV CD8^+^ specificities in a proportion of patients with CHB by blocking PD-1 pathway apparently shows that there exists other mechanisms other than PD-1/PD-L1 that may contribute to T-cell exhaustion. Although the underlying mechanism involved in the upregulation of PD-1 remains to be explored, it has recently been implicated that in chronic LCMV infection mice, T-bet represses expression of PD-1 and sustains exhausted CD8^+^ T cells.^23^ Accordingly, reduced expression of PD-1 on HBV-specific CD8^+^ T cells with high levels of T-bet was found to support the key role for T-bet in regulating inhibitory molecules of exhausted T cells during chronic HBV infection,^36^ which may account for T-bet deficiency as an important mechanism behind chronic infection.

With the reversed immune dysfunction of HBV infection, there can be no argument that the anti-PD-1 monoclonal antibody (mAb) might be a good therapeutic candidate for chronic HBV infection. However, the reasons why partial responses of T cells can be restored following PD-1 pathway blockade and or blockade of multiple inhibitory molecules may be complicated. Because persistent expression of HBV antigens can be observed in the hepatocytes of immunocompromised mice after hydrodynamic injection of HBV plasmid DNA,^37^ and the genetic background of recipients, which correlates with the strength of response against HBV antigens, it also determines the outcome after hydrodynamic injection.^38^ And, the participation of third signal cytokine IL-12 was able to augment the capacity of HBV-specific CD8 T cells to produce effector cytokines upon stimulation accompanied by the downregulation of PD-1 and an increase in the transcription factor T-bet.^36^ Consistent with this, one more research confirmed that IL-12 selectively induced the phosphorylation of STAT4 in T-bet^+^ CD8 T cells,^39^ and hence it can be inferred that genetic factor, antigen load and transcriptional factors including T-bet, as well as cytokine pathways such as IL-12, all contribute to the partial recovery of the function of exhausted T cells.

## CTLA-4 (CD152)

Coinhibitory signals have been proposed to drive the T-cell exhaustion during chronic viral infections.^25^ One of the well-elaborated coinhibitory molecules is CTLA-4 (CD152). T-cell activation requires a T-cell receptor (TCR)-mediated signal accompanied by a costimulatory signal through the CD28 molecule. On the contrary, inhibitory CD28-B7 family member CTLA-4 is generally induced upon TCR engagement, which hampers CD28-dependent T-cell activation^40^ by cell-intrinsic mechanisms such as delivering inhibitory signals that induce cell cycle arrest and prevent the production of IL-2^41, 42^ or by cell-extrinsic mechanisms including affecting Treg cells.^43, 44^ Studies of the role CTLA-4 in chronic infections have diverse results. Whereas in chronic murine LCMV infections, CTLA-4 was shown not to be involved in CD8^+^ T-cell exhaustion,^18^ but CTLA-4 can regulate the murine immune response to *Trypanosoma cruzi*^45^ and *Leishmania* infection.^46^ In patients with CHB, HBV-specific CD8^+^ T cells were verified to have an increased propensity to express CTLA-4 strongly correlating with viral load; accordingly, blockage of CTLA-4 was able to increase the proliferation of IFN-*γ*-producing HBV-specific CD8^+^ T cells in the periphery and liver tissues. Simultaneously, it has been demonstrated that suppression of human CTLA-4 mRNA in lymphocytes by using RNA interference *in vitro* induced the upregulation of IFN-*γ* and IL-2.^47^ The possible mechanisms of CTLA-4-mediated immunoregulation in chronic HBV infections may be owing to the identification of CTLA-4 gene polymorphisms related to HBV viral clearance.^48^ Furthermore, CTLA-4-mediated exhaustion may be Bim-dependent (a proapoptotic mediator) based on the fact that CTLA-4^hi^ HBV-specific CD8^+^ T cells displayed highest intracellular levels of Bim, as well as in most CHB patients without evidence of liver inflammation, suppression of the CTLA-4 receptor reduced the expression of Bim.^49^ Besides, CTLA-4 was shown to increase T-cell motility and override the TCR-induced stop signal required for stable conjugate formation between T cells and antigen-presenting cells (APCs).^50^ In spite of its well-documented inhibitory function on exhausted T cells, CTLA-4 blockade can also exacerbate autoimmune disease, which restrains the safety and activity of anti-CTLA-4 Abs in primates.^51^ Thus, restoration of the impaired T-cell response by blockage of CTLA-4 is an attractive strategy for complete eradication of HBV infection, and further investigation is urgently needed to comprehend the immunoregulation of inhibitory receptors, which is designed to address related problems and improve the clinical outcome of our current standard treatment. In addition to HBV infection, more advances have been achieved in the role of anti-CTLA-4 treatment in other chronic viral infection, and cancers such as tremelimumab as mAb against CTLA-4 has been certified with safe antitumor and anti-viral activity in the clinical trials of hepatocellular carcinoma (HCC) and HCV infection,^52^ together with the ability to induce tumor responses in a subset of patients with metastic melanoma^53, 54^ and colorectal cancer.^55^ In a phase 3 study, ipilimumab (another CTLA-4-blocking mAb) has demonstrated an improvement in overall survival among patients with metastatic melanoma.^56^ Studies on HIV infection have revealed that, in contrast to PD-1, CTLA-4 is highly expressed on HIV-specific CD4^+^ T cells, but absent on HIV-specific CD8^+^ CTLs. *In vitro* blockade of CTLA-4 augmented HIV-specific CD4 T-cell proliferation and function.^57, 58^ Collectively, the above findings herald a new field of immunotherapy for the chronic HBV infection by blocking CTLA-4.

## CD244/CD48

Although PD-1/PD-L1 is the best-characterized inhibitory receptor correlated with exhaustion, there are several other receptors displayed to impair T-cell responses during chronic HBV infections. CD244, also known as 2B4, was firstly described as an inhibitory receptor highly expressed on exhausted CD8^+^ T cells in line with PD-1 in chronic LCMV infection and chronic HBV infection in mice.^27, 59^ Subsequently, Jung *et al.*^26^ addressed CD244 as an important inhibitory molecule on HBV virus-specific CD8^+^ T cells by providing evidence that in chronic HBV-infected patients, these cells expressed higher levels of CD244 coincidence with an increased expression of PD-1 both in the peripheral blood and hepatic tissue compared with acute and resolved infection. Results from this study also revealed that CD244 or its ligand CD48 blockade may recover T-cell proliferation, cytokine production and cytotoxicity of exhausted HBV-specific CD8^+^ T cells in chronic infection but not in acute and resolved infection. The above observations indicate that CD244 may contribute to T-cell exhaustion independently or act in corporation with other inhibitory molecules in chronically infected HBV patients. Despite the lack of detailed mechanism of inhibitory function and interaction of CD244/CD48, further studies will be necessarily focusing on underlying principles, and apparently, T-cell restoration by blocking CD244/CD48 might have important implications as another potential target for immunotherapy in chronic HBV infections.

## Tim-3/Galectin-9

Tim-3 is a negative regulatory molecule expressed on T cells, which is bound by galectin-9 to drive the death of Th-1 cells and promote peripheral tolerance.^60^ Previous studies in patients persistently infected with HCV or HIV have shown that Tim-3 contributes to the dysfunction of CD8^+^ T cells in persistent viral infections.^61, 62^ More recently, Nebbia *et al.*^63^ found that in HBV-infected individuals receiving anti-viral treatment, Tim-3 expression was significantly increased on the exhausted HBV-specific CD8^+^ T cells compared with the overall CD8^+^ T cell within the same patient.^63^ Additionally, patients who had resolved HBV infection had conspicuously lower levels of Tim-3, indicating a correlation between Tim-3 and clinical outcome. With regard to its influence on the function of T cells, HBV peptide-specific CD8^+^ T cells with high expression of Tim-3 had an impaired ability to produce IFN-*γ* and TNF-*α*. By blocking Tim-3/galectin-9 interactions *in vitro* in these patients, expansion of HBV-specific CD8^+^ T cells was enhanced, which were able to produce cytokines and mediate cytotoxicity.^63^ Results from Tim-3/galectin-9 blockade *in vitro* demonstrated that the effect on HBV-specific CD8^+^ T cells resulted from Tim-3/galectin-9 interactions in patients with CHB were biased towards deletion instead of inactivation, in line with previous reports.^61, 62^ Besides, enhanced cytotoxicity was also observed in PBMCs or NK cells in CHB patients treated with the Tim-3 blockade *ex vivo*.^64^ Importantly, Tim-3 was evidenced to have a non-redundant role in chronic HBV-infected patients similar to PD-1; thus, Tim-3 and PD-1 may form distinct populations or overlapping subsets. As a natural ligand for Tim-3, the action of galectin-9 on T-cell exhaustion has become nonnegligible with the property that galectin-9 being preferentially expressed in the liver close to the sinusoids.^65^ It is postulated that galectin-9 may act upon Tim-3-expressing HBV-specific CD8^+^ T cells during inflammatory infiltration and be responsible for their deletion and inactivation. A recent study has already reported high galectin-9 expression on Kupffer cells in HCC islets, but not in the adjacent tissues.^66^ Observations of liver biopsies from patients with CHB also showed strong staining of galectin-9 in Kupffer cells, together with considerably elevated levels of galectin-9 in the circulation of patients with HBV-related liver inflammation.^63^ Combined with the blockade result mentioned before, galectin-9 was proved to contribute to the inhibition and deletion of T cells in CHB patients as they infiltrate the HBV-infected liver. Although the ability of galectin-9 to induce T-cell death via Tim-3 is well described, the inherent mechanisms remain poorly explicit, coupled with the effect that galectin-9 may negatively regulate T cells via the induction of other regulatory populations such as Tregs.^67, 68^ Therefore, further investigation on the exact functional mechanism of galectin-9 on exhausted T cells will be necessary and potential application of Tim-3/galectin-9 blockade needs to be taken into consideration with concern to the immunotherapy against persistent HBV infection.

## Functional Change and Potential Mechanism of CD8^+^ T-cell Exhaustion in HBV Infection

The HBV-specific CD8^+^ T cells exert their ability of viral clearance mainly by the production of anti-viral cytokines such as IFN-*α*/*γ* and granzyme/perforin.^69^ Aberrant functional profile was discovered in exhausted CD8^+^ T cells from CHB patients, namely reduced IL-2 production and preserved production of the proinflammatory cytokines including IFN-*γ* and TNF-*α*.^70^ As IL-2 is required to drive CD8^+^ T cells to proliferate in the absence of CD4^+^ T cells,^71^ the disruption in IL-2 production evidently impacted the expansion of this small population of CD8^+^ T cells during chronic HBV infection. As proinflammatory cytokines, IFN-*γ* and TNF-*α*, were both able to promote hepatitis through distinguished mechanisms,^72, 73, 74^ the maintenance of IFN-*γ* and TNF-*α* production in exhausted T cells could drive nonspecific immunoresponse to persistent inflammation. Contrarily, more studies support the idea that during chronic HBV infection, there is a lack of antigen-specific IFN-*γ* production from virus-specific CD8^+^ T cells.^39^ Another study demonstrated that decreased IL-21 secreted from HBV-specific CD4^+^ T cells partly contributes to the exhaustion of specific cytotoxic CD8^+^ T-cell response in chronic HBV infection. These findings provide clues for rational design of new therapeutic strategy against chronic HBV infection.^75^

## Characteristics and Mechanism of CD4^+^ T-cell Exhaustion in Chronic HBV Infection

Similar to T helper (Th) cells, CD4^+^ T cells are the dominant regulators of the CD8^+^ T-cell-mediated HBV response and clearance.^76^ Furthermore, during HBV infection, CD4^+^ T cells were shown to have essential roles on effector and memory CD8^+^ T-cell responses.^77, 78^ Other than high levels of virus antigen, a lack of CD4^+^ Th cells was considered to be one of the major causes resulting in the exhaustion of specific CD8^+^ T cells^12^ because of multiple properties; CD4^+^ T cells can activate professional APCs such as DCs via interaction between CD40 and CD40 ligand, or produce some cytokines (IL-2 and IL-21) and chemokines, which are responsible for introducing the naive T cells to the sites of priming in secondary lymphoid organs as well as activated T cells to the location of the infection.^79, 80, 81^ Therefore, under the condition of chronic viral infection, CD8^+^ T cells succumbed to exhaustion seem more severe without the help of CD4^+^ T cells.^12^

Although CD4^+^ T-cell exhaustion has been recently shown to develop in more and more studies,^82, 83, 84^ a comprehensive illustration and mechanism of CD4^+^ T-cell exhaustion in chronic HBV-infected patients remains less well understood. With a newly established DRB1*01-restricted MHC class II tetramer, Jung *et al.* defined a sustained expression of PD-1 on CD4^+^ T cells in chronic HBV patients, companied by low expression of other inhibitory receptors including CTLA-4, TIM-3, KLRG1 (killer cell lectin-like receptor subfamily G member 1) and CD244.^85^ Because these inhibitory molecules were displayed less than those presented by exhausted CD8^+^ T cells in chronic infection,^26, 27^ it is probably speculated that exhausted CD4^+^ T cells were less similar to CD8^+^ T cells. In the same study, the authors also revealed that neutralization of PD-L1/2 was able to improve the ability of CD4^+^ T cells to produce Th-1 cytokines including IFN-*γ*, IL-2 and TNF-*α*, with enhanced T-cell proliferation in treated patients with successful viral control. Although in the study mentioned previously, TIM-3 was evidenced to be highly expressed on exhausted HBV-specific CD4^+^ T cells,^63^ Jung *et al.*^85^ suggested that blocking CTLA-4 or TIM-3 singly failed to reactivate CD4^+^ T-cell function, which is in contrast to that of exhausted CD8^+^ T cells by blocking TIM-3/galectin-9 interactions, HBV-specific CD8^+^ T-cell responses can be rescued complementary to PD-1 pathway inhibition,^63^ supporting that inhibitory molecule TIM-3 may exert different suppressive effects on CD4^+^ and CD8^+^ T cells in chronic HBV infection. These findings provide primary evidence for the exhaustion of CD4^+^ T cells by defining the inhibitory phenotype and the changes of CD4^+^ T-cell function during chronic HBV infection, and provide new insights into the mechanisms underlying CD4^+^ T-cell exhaustion, as well as novel immunological approaches for prospective therapeutic strategy for persisting HBV infection. Observations on the alteration of exhausted CD4^+^ T cells in other viral diseases such as HCV and HIV support the theory that CD4^+^ T cells have important role in chronic viral infection. Similar to inhibitory molecules, PD-1 and CTLA-4 can regulate the expansion and restoration of HCV-specific CD4^+^ T cells in patients with chronic HCV infection,^86^and the loss of proliferative function followed by deletion of HCV-specific CD4^+^ T cells is closely related to HCV viremia.^87^ Upregulation of inhibitory molecules including PD-1 and CTLA-4 were also observed on HIV-specific CD4^+^ T cells correlating with disease progression and defining a reversible immune dysfunction,^58, 88^ which will help to interpret the related phenotype and mechanism found in HBV-infected patients.

## Suppressive cytokines and CD4^+^ Treg Cells Contribute to Exhaustion of T Cells in Chronic HBV Infection

Except for those intrinsic pathways that may contribute to the regulation of T-cell exhaustion, extrinsic pathways such as immunosuppressive cytokines and Treg cells may also participate in the T-cell exhaustion in chronic HBV. It is known that murine Kupffer cells in the liver constitutively express IL-10 and TGF-*β*, which induce the tolerance of liver-infiltrating lymphocytes.^89^ Specifically, in HBV infection, IL-10 is negatively associated with the effect of HBV.^90, 91^ In addition, specific polymorphism of IL-10 has been correlated with increased severity of chronic HBV infection.^92^ Similar to immunosuppressive cytokines, TGF-*β* is possessed with negative effects on virus-specific CD8^+^ T-cell function. And, both IL-10 and TGF-*β* can limit the proliferative and survivable abilities of T cells, thereby attenuating viral control.^91^ Mechanically, blockade of TGF-*β* led to an enhanced secretion of IFN-*γ* by HCV-specific CD8^+^ T cells,^93^ suggesting an important role of TGF-*β* in T-cell exhaustion. Although it is not defined whether TGF-*β* is the universal determinants of exhaustion during persistent HBV infections, immunotherapeutic targeting IL-10 and TGF-*β* in CHB may provide an additional therapeutic intervention, which needs further exploration. Interestingly, the functions of CD4^+^ Treg cells on HBV-specific T cells have been widely described, but controversy exists in the impact of CD4^+^ Treg on T-cell exhaustion. On the one hand, several studied have suggested that patients with chronic HBV infection exhibited a significantly high frequency of circulating CD4^+^ Treg cells as opposed to that of controls and resolved HBV infection that inhibit the proliferation of HBV-specific CD8^+^ T cells.^94, 95^ On the other hand, it has been shown in another study that there was no markedly increased number of circulating CD4^+^ Tregs from patients with chronic HBV infection compared with those patients with resolved infection; moreover, these cells in patients with different profiles of HBV infection did not show remarkable functional difference,^96^ signifying irrelevance between chronic HBV infection and these inhibitory cell subset. Shortly afterwards, one more study identified that chronically HBV-infected patients with high viral loads had a higher proportion of Treg cells in the liver, but not in blood, and these intrahepatic Tregs were phenotypically distinct from peripheral blood Tregs.^97^ Taken together, these results suggest that CD4^+^ Tregs have a role in T-cell exhaustion during chronic HBV infection; however, the mechanisms that mediate the regulatory effect of CD4^+^ Tregs are still controversial. The combination therapy of inhibitory receptor and IL-10, TGF-*β* or Treg cells in chronic HCV patients achieved highly heterogeneous results,^98^ proving that individualized treatment should be considered in the design of future immunomodulatory therapies.

## Conclusion and Future Perspectives

**Figure 3:**
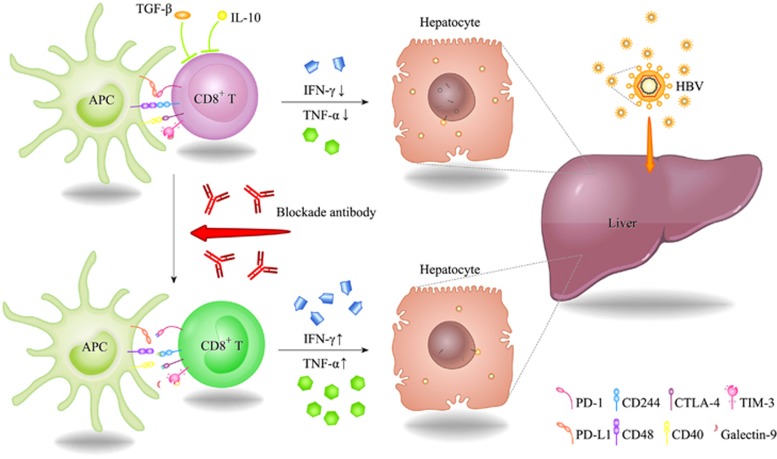
Immunotherapy for HBV treatment achieved by restoration of exhausted T cells. Exhausted T cells are subject to a number of signaling pathways through which inhibitory receptors can transmit suppressive signals to inhibit functional and proliferative responses during chronic HBV infection. Existing data support that inhibitory receptors including PD-1, CTLA-4, TIM-3 and CD244 all take part in the immunoregulatory mechanism of T-cell exhaustion. Therefore, the restoration of exhausted T-cell function appears effective by either blocking one of these inhibitory receptors or simultaneously blocking several inhibitory molecules. Besides, blocking inhibitory cytokines such as IL-10 or TGF-*β* are also considered to be a beneficial approach to modulate the function of T cells. After antibody blockade, the ability of T cells to proliferate and secrete cytokines can be restored in chronic HBV-infected patients. Based on these observations, immunotherapeutic approaches deserve better exploration for anti-viral treatment of HBV patients

The ability to clear HBV after infection is associated with a strong virus-specific T-cell response, and the function of exhausted virus-specific T cells during chronic infections is often characterized with varying degrees of damage; thus, the restoration of exhausted T-cell function is of great importance to eliminate infiltrating virus. Coinhibitory pathways such as PD-1, CTLA-4 and Tim-3 have been proved to be upregulated in chronic viral hepatitis and critical in hepatic tolerance, contributing to the failure of T-cell response to hepatic pathogens such as HBV, which can be regarded as novel indicators of T-cell immune function in patients with chronic HBV infection. Moreover, therapeutic interventions targeting exhausted T cells through the blockade of these suppressive pathways might have important implications, since by blocking inhibitory molecules, the function of exhausted T cells can be restored in chronic HBV-infected patients,^49, 64, 99^ as summarized in Table 2 and Figure 3. However, because of the individual susceptibility to different inhibitory molecules, the blockade of single inhibitory pathways shows a broad variability in the recovery of T-cell response to persistent viral stimulation, such as defects in CD8^+^ T cells remain after PD-1 pathway blockade.^18^ These observations might be explained by the synergy and redundancy of multiple inhibitory receptors on T cells, because a combined blockade of PD-1 and lymphocyte activation gene-3 synergistically improved T-cell function.^27^ Altogether, these results indicate that T-cell exhaustion is regulated by a complex network of coexpressed inhibitory receptors acting synergistically and redundantly. Hence, a better understanding of diverse inhibitory receptors in the regulation of exhausted T cells will pave the way for the development of more effective immunotherapeutic and prophylactic strategies for the treatment of chronic infectious diseases.

Recently, several studies have provided insights into the transcriptional mechanisms behind T-cell exhaustion in chronic infection. T-bet has been demonstrated to repress directly the expression of inhibitory receptor PD-1 and sustain virus-specific CD8^+^ T-cell responses during chronic infection with its expression downregulated in exhausted CD8^+^ T cells in response to persisting antigen.^23^Besides, in HBV and HCV infection, related findings support a critical role of T-bet for viral clearance and consider T-bet deficiency was more characteristic of chronic evolving infection.^39^ Similar to T-box transcription factor, the role of eomesodermin (Eomes) in chronic infection is controversial, as some researchers found that Eomes expression was upregulated in exhausted CD8^+^ T cells during chronic infection,^100, 101^ whereas others consider that Eomes might compensate for the lack of T-bet in HBV and HCV infection. Although there was no obvious upregulation of Eomes,^39^it still needs further exploration. The transcriptional repressor Blimp-1 (Pdrm1) was initially recognized for its role in regulating terminal differentiation of B cells, but recently it has been identified to regulate T-cell responses and the generation of CD8^+^ effector memory T cells.^102^ During chronic viral infection, Blimp-1 repressed normal memory CD8^+^ T-cell differentiation and promoted high expression of inhibitory receptors similar to PD-1, whereas high expression of Blimp-1 fostered aspects of CD8 T-cell exhaustion.^59^ One regulator of Blimp-1 is IL-21, as IL-21 signaling can induce Blimp-1 expression in preactivated CD8^+^ T cells; however, there must be other regulators of Blimp-1 that involve in the exhaustion of T cells in chronic viral infection.^103^ Although similar transcriptional mechanisms were found behind CD4^+^ and CD8^+^ T cells, there is obvious heterogeneity in TF expression between these two subsets of exhausted T cells. As a consequence, future studies may investigate in detail the distinct transcriptional pathway involved in the immunoregulation of T-cell exhaustion in chronic virus infection, providing different molecular controls and therapeutic targets to treat chronic HBV infection.

## Figures and Tables

**Table 1:** Clinical manifestation and immunological courses of chronic HBV-infected patients

**Status**	**Immunological characteristics**	**Immunological result**	**Clinical manifestation**	**References**
Immune clearance	IL-12↑↑, IL-18↑↑, IFN-*γ*↑, IFN-*α*↑, IL-10↓,	Inflammatory response, cell infiltration (T cell, NK, NKT cell and monocytes), complete or partial virus deletion	Active hepatitis B, liver damage An elevated level of ALT	^1, 3, 4, 5, 104, 105^
Immune tolerance	IL-10↑, TGF-*β*↑, PD-1↑, CTLA-4↑, Treg↑	Exhaustion, apoptosis persistent HBV replication	Low-grade inflammation Normal or low level of ALT	^1, 2, 3, 14, 105^

Abbreviations: HBV, hepatitis B virus; IL, interleukin; IFN-*γ*, interferon-*γ*; TNF-*α*, tumor necrosis factor-*α*; TGF-*β*, transforming growth factor-*β*; PD-1, programmed cell death-1; CTLA-4, cytotoxic T-lymphocyte-associated antigen-4; Tregs, T-regulatory cells, NK cells, natural killer cells; ALT, alanine aminotransferase.

**Table 2:** Characteristics and potential immunotherapeutics of T-cell exhaustion during chronic HBV infections

	**CD8**^**+**^ **T cells**	**CD4**^**+**^ **T cells**	**References**
Characteristics	Decreased HBV-specific CD8^+^ T cells; impaired cytotoxicity (IL-2, IFN-*γ* and TNF-*α*); increased inhibitory receptors including PD-1, TIM-3, CTLA-4 and 2B4	Impaired proliferation without obvious defect in Th-1-type cytokines; high PD-1 and low expression of CTLA-4, TIM-3, KLRG1 and 2B4	^1, 9, 11, 25, 26, 27, 28, 106, 107^
Evidence for immunotherapy	Blockade of the PD-1/PD-L1 pathway restored proliferation and function of HBV-specific CD8^+^ T cells; blockade of CD244 or its ligand CD48 may recover proliferation, cytokine production and cytotoxicity of exhausted HBV-specific CD8^+^ T cells; blockade of CTLA-4 was able to increase the proliferation of IFN-*γ*-producing HBV-specific CD8^+^ T cells; by blocking Tim-3/galectin-9 interactions, expansion of HBV-specific CD8^+^ T cells was enhanced, which were able to produce cytokines and mediate cytotoxicity	PDGF-BB inhibited CD4^+^ T-cell proliferation; antibody specific for the PDGF-B chain was able to reduce the development of liver fibrosis; neutralization of PD-L1/2 was able to improve the ability of CD4^+^ T cells to produce Th-1 cytokines	^11, 26, 35, 47, 49, 51, 61, 62, 63, 106, 107^

Abbreviations: HBV, hepatitis B virus; IL-2, interleukin-2; IFN-*γ*, interferon-*γ*; TNF-*α*, tumor necrosis factor-*α*; PD-1, programmed cell death-1; PD-L1, programmed death-ligand 1; CTLA-4, cytotoxic T-lymphocyte-associated antigen-4; TIM-3, T-cell immunoglobulin domain and mucin domain 3; 2B4, CD244; KLRG1, killer cell lectin-like receptor subfamily G member 1; PDGF-BB, platelet-derived growth factor-BB.

## Abbreviations

HBV: hepatitis B virus
HCV: hepatitis C virus
LCMV: lymphocytic choriomeningitis
PD-1: programmed cell death-1
PD-L1: programmed death-ligand 1
CTLA-4 (CD152): cytotoxic T-lymphocyte-associated antigen-4
TIM-3: T-cell immunoglobulin domain and mucin domain 3
HCC: hepatocellular carcinoma
IFN-*γ*: interferon-*γ*
TNF-*α*: tumor necrosis factor-*α*
IL-2: interleukin-2
IL-10: interleukin-10
IL-21: interleukin-21
TGF-*β*: transforming growth factor-*β*
Bim: Bcl2-interacting mediator
TCR: T-cell receptor
PBMC: peripheral blood mononuclear cell
Treg: T-regulatory cell
NK cell: NK cell
APC: antigen-presenting cell
DC: dendritic cell
KLRG1: killer cell lectin-like receptor subfamily G member 1
PDGF-BB: platelet-derived growth factor-BB
NFAT: nuclear factor of activated T cell
T-bet: T-box transcription factor
Blimp-1: PR domain zinc-finger protein 1
